# Eicosapentaenoic Acid (EPA) and Docosahexaenoic Acid (DHA) in Muscle Damage and Function

**DOI:** 10.3390/nu10050552

**Published:** 2018-04-29

**Authors:** Eisuke Ochi, Yosuke Tsuchiya

**Affiliations:** 1 Faculty of Bioscience and Applied Chemistry, Hosei University 3-7-2, Kajino, Koganei, Tokyo 184-8584, Japan; 2 Faculty of Modern life, Teikyo Heisei University 4-22-2, Nakano, Tokyo 164-8530, Japan; y.tsuchiya@thu.ac.jp

**Keywords:** omega3, n-3, unsaturated fatty acids, eicosapentaenoic acid, docosahexaenoic acid, muscle damage, muscle hypertrophy, muscle strength, neuromuscular function

## Abstract

Nutritional supplementation not only helps in improving and maintaining performance in sports and exercise, but also contributes in reducing exercise fatigue and in recovery from exhaustion. Fish oil contains large amounts of omega-3 fatty acids, eicosapentaenoic acid (EPA; 20:5 n-3) and docosahexaenoic acid (DHA; 22:6 n-3). It is widely known that omega-3 fatty acids are effective for improving cardiac function, depression, cognitive function, and blood as well as lowering blood pressure. In the relationship between omega-3 fatty acids and exercise performance, previous studies have been predicted improved endurance performance, antioxidant and anti-inflammatory responses, and effectivity against delayed-onset muscle soreness. However, the optimal dose, duration, and timing remain unclear. This review focuses on the effects of omega-3 fatty acid on muscle damage and function as evaluated by human and animal studies and summarizes its effects on muscle and nerve damage, and muscle mass and strength.

## 1. Introduction

Nutritional supplementation is important for improving and maintaining performance in exercise interventions. However, the optimal dose, duration, and timing remain unclear in addition to detailed mechanism. Omega-3 fatty acids include of eicosapentaenoic acid (EPA; 20:5 n-3) and docosahexaenoic acid (DHA; 22:6 n-3), which are mainly contained in fish oil. Omega-3 fatty acid first garnered attention when it was found that the heart disease rate was markedly low in the Greenland Eskimos, who consumed large amounts of these fatty acids [1,2]. From that time, many studies have been published, making it widely known that omega-3 fatty acids are effective for improving cardiac function and blood as well as lowering blood pressure and improving depression and cognitive function [3,4,5,6,7,8,9,10]. In terms of involvement with exercise performance, EPA and DHA are known in particular to improve fatigue recovery and endurance performance, as well as maintain immune function [5,11,12]. In addition, exhaustive or unaccustomed exercise causes muscle fatigue and delayed onset muscle soreness (DOMS), resulting in decreased exercise performance [13,14,15]. At the same time, oxidative stress and inflammatory reaction occur [16,17]. Many studies have investigated these topics as EPA and DHA are anticipated to be effective against such reactions [18,19,20,21]. This review focuses on the effects of EPA and DHA on muscle damage and function as evaluated by human and animal experiments. Specifically, we summarize based on past studies these effects on: (1) muscle and nerve damage; and (2) muscle mass and strength.

## 2. EPA and DHA for Muscle and Nerve Damage

Decreased muscle strength, DOMS, muscle swelling, and limited range of motion occur after eccentric contractions (ECCs) [13,16,22]. DOMS peaks 1–3 days after exercise [13,16,20] and has an uncomfortable and negative effect for continuing exercise and training. Because DOMS can also cause decrease muscle strength and reduce flexibility as well as lower exercise performance, the prevention and alleviation of muscle damage following ECCs are important issues. Muscle damage caused by ECCs is thought to be caused by micro-damage for muscle fibers, inflammatory response, and oxidative stress [23,24,25]. Since many researchers have studied the effects of omega-3 fatty acids on these phenomena [18,20,26,27,28,29,30,31,32,33], we summarize the findings of previous studies for each topic below.

### 2.1. Muscle Strength Deficit

**Figure 1:**
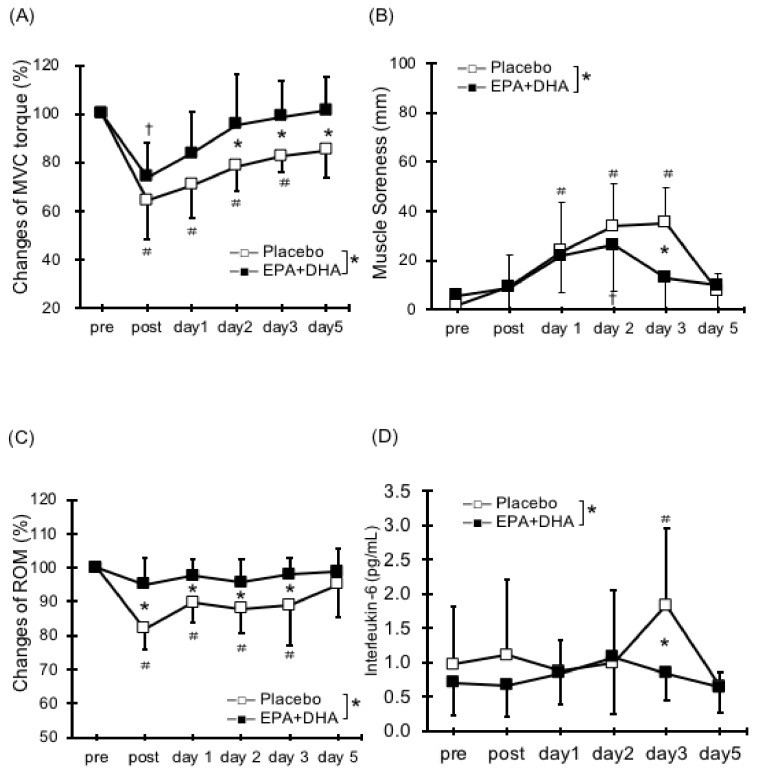
Changes (means ± SD) in: maximal voluntary isometric contraction (MVC) torque (**A**); muscle soreness (**B**); range of motion (ROM) (**C**); and Interleukin (IL)-6 (**D**), before (pre), immediately after (post), and 1, 2, 3, and 5 days after eccentric contractions in EPA group and placebo. * (*p* < 0.05); significant difference between groups, † (*p* < 0.05); significant difference from pre-exercise value in EPA group, # (*p* < 0.05); significant difference from pre-exercise value in placebo group (data are from Tsuchiya et al., 2016).

There are few reports about the effects of EPA and DHA supplementation on decreased muscle strength caused by ECCs (Table 1). Houghton and Onambele [34] evaluated resistance exercise for the lower limbs after 0.36 g/day ingestion of EPA over three weeks and demonstrated no significant differences in muscle strength reduction between the EPA and placebo. In addition, the untrained subjects with only 2.0 g/day of DHA for four weeks did not differ in muscle strength after 60 ECCs in elbow flexors [35]. Lenn et al. [36] tested thirteen untrained men (age: 22.7 ± 3.92, BMI: 24.1 ± 2.7) and nine untrained women (age: 24.5 ± 5.47, BMI: 23.6 ± 5.3) randomly assigned to fish oil group (*n* = 7), wheat flour (soy control) group (*n* = 7), or isoflavone group (*n* = 8). They found that ingestion of 0.287 g/day of EPA and 0.194 g/day of DHA for 30 days did not reduce the decrease in muscle strength after 50 ECC in elbow flexors. Based on the results of these studies, we applied 30 ECCs in elbow flexors after the long-term intake of both 0.6 g/day of EPA and 0.26 g/day of DHA for eight weeks. We found that ingestion of EPA and DHA causes an inhibition in torque deficit (17%) (Figure 1A) [20]. 

Taken together, the effects of EPA and DHA supplementation on strength loss after ECC have been controversial. However, it was reported that the compensation of EPA and DHA concentration into the human myocardium requires 30–60 days of ingestion [37] to achieve the inhibition of muscle strength deficit by EPA and DHA intake, it appears important to ingest over an eight-week period.

### 2.2. Delayed Onset Muscle Soreness (DOMS)

Many studies have reported on the effects of EPA and DHA intake on DOMS [20,28,29,33]. The supplementation of 0.324 g/day of EPA and 0.216 g/day of DHA daily for 30 days inhibited DOMS after 40-min bench stepping [31]. Jouris et al. [27] reported the effect of 2.0 g/day of EPA and 1.0 g/day of DHA for two weeks on DOMS after ECCs at 120%1 repetition maximum (RM) until exhaustion using dumbbells. They observed decreased DOMS in the EPA and DHA group. The finding is consistent with our work (0.6 g/day of EPA and 0.26 g/day of DHA for eight weeks; Figure 1B) [20]. Meanwhile, it has been shown that the ingestion of either DHA or EPA had no effect on DOMS [30,35]. Although the dose and period are different from the previous studies, we suggest that the ingestion of both EPA and DHA may be important in reducing DOMS and has a synergistic effect on DOMS attenuation, especially at the ratio of approximately 2:1. Otherwise, DOMS after ECCs for knee flexors did not change with or without EPA and DHA supplement (1.3 g/day of EPA and 0.3 g/day of DHA for six weeks) [38]. Thus, these findings suggest that EPA and DHA supplementation have a certain effect to inhibit DOMS by ECCs, but it may differ depending on the dose of EPA and DHA and the exercise site (Table 2).

### 2.3. Range of Motion

There have only been few studies on the effects of EPA and DHA on range of motion (ROM) reduction after ECCs [20,28,29,31,36] (Table 3). EPA and DHA attenuated ROM reduction after 40-min bench stepping [31]. Our study also found that 0.6 g/day of EPA and 0.26 g/day of DHA for eight weeks inhibited the reduction of ROM (Figure 1C) [20,29]. Contrarily, two other studies reported that there were no effects on ROM reduction following ECCs of elbow flexors [28,36]. Thus, no fixed consensus has been reached regarding the effects of EPA and DHA intake on ROM restriction. Because decreased flexibility may contribute to reduced exercise performance and increase the risk of injuries, more studies are needed on the effects of EPA and DHA ingestion on flexibility, including muscle stiffness.

### 2.4. Swelling (Circumference and Cross-Sectional Area)

Excessive muscular exercise including ECCs causes muscle swelling [16]. Regarding the relationship between EPA and DHA ingestion and muscle swelling, Taribian et al. [31] found that EPA and DHA inhibited the increases in thigh circumference after bench stepping. Other studies did not find differences between treatment and placebo group on femoral and upper arm circumference [27,33]. It appears that above results in previous studies may have been caused by the variation and precision in tape measurement. Therefore, we evaluated cross sectional area of elbow flexors using ultrasound [29]. Our results indicated that, although there was no significant difference, supplementation of EPA and DHA caused a tendency for inhibition of increased cross-sectional area (CSA). More precise CSA evaluation using magnetic resonance imaging (MRI) needs to be conducted [13] (Table 4).

### 2.5. Serum Cytokines and Muscle Damage Markers

Regarding serum inflammatory markers after ECCs, EPA and DHA supplement inhibit elevated tumor necrosis factor-α (TNF-α) and interleukin-6 (IL-6), which are inflammatory markers in the blood [32]. As described above, we demonstrated that EPA and DHA ingestion reduced the levels of IL-6 (Figure 1D) [20]. Furthermore, as a result of ingesting 0.36 g/day of EPA for 3 weeks, it has been reported that rises in IL-6 were inhibited after four types of resistance training for the lower limbs [34]. Other studies have indicated that EPA and DHA ingestion can inhibit the elevation in levels of IL-6 and TNF-α after ECCs and running exercise [26,30,32,35,39]. Thus, EPA and DHA supplementation has a positive role to inhibit inflammatory response following eccentric exercise. Since limited ROM following ECC has been attributed to inflammatory response within myofibrils leading to increases in passive stiffness [40], it assumes that the inflammatory response after ECCs is reduced by the EPA and DHA and thereby related to inhibition of ROM reduction and swelling. More detailed discussion of the inflammation, including oxidative stress and immune function can refer to earlier reviews [21] (Table 5). 

Serum creatine kinase (CK) and myoglobin (Mb) are elevated by ECCs, which are typical skeletal muscle damage markers [16]. No consensus has been achieved regarding the inhibition of increases in CK and Mb with EPA and DHA ingestion. EPA and DHA for 30 days have a effective role to reduce the CK and Mb after bench stepping [32]. Meanwhile, after 1.3 g/day of EPA and 0.3 g/day of DHA were ingested daily for 6 weeks, there were no changes in CK rises following knee bending exercise [38]. We confirmed that there were no differences between groups in CK or Mb after ECCs (0.6 g/day of EPA and 0.26g g/day of DHA for 8 weeks) [20]. With regards to the single intake for DHA, 2 g per day for 4 weeks caused an inhibition for CK elevation [35], while 0.8 g/day for 2 weeks showed no change [30]. One possible reason for these inconsistent results for CK and Mb response is differences in exercise type and individual differences between subjects. It may be necessary to develop new muscle damage-related marker with little variation, and to conduct verifications using it.

### 2.6. Neuromuscular Damage

Previous studies have shown that nerve dysfunction is also induced by ECCs [25,41]. Nerve conduction velocity (NCV) decreased by 12–24% 1–2 days after ECCs, suggesting that this prolonged NCV was related to muscle strength deficit [25]. The relationship between NCV and EPA and DHA has been investigated in an animal model [42]. Gerbi et al. [42] used diabetic rats to investigate the relationship among omega-3 intake, NCV, endoneurial edema and axonal degeneration. They reported that EPA and DHA ingestion inhibited decreases in NCV in diabetic rats [42]. They proposed that EPA and DHA ingestion prevented membrane alteration and having Na, K-ATPase gene transcription effects [42]. Based on their results, we investigated whether EPA and DHA intake affected post-ECCs NCV in humans [29]. Our results indicated that the supplementation of EPA and DHA daily for eight weeks inhibited musculocutaneous NCV latency following ECCs. This observation can be assumed that EPA and DHA protects neuromuscular function, but there is only one study that examined the effect of EPA and DHA on nerve system in human. Hence, further evidence is required around this field.

## 3. EPA and DHA for Muscle Mass and Function

### 3.1. Muscle Mass

Maintaining skeletal muscle mass is important not only for athletes but also for common people from the viewpoint of sarcopenia. In previous studies, muscle hypertrophy has been evaluated by CSA of muscle, muscle thickness, growth factors and hormones, and protein metabolism (protein synthesis rate and insulin signaling pathway) [43,44,45,46,47,48]. Interestingly, some findings have indicated that the ingestion of EPA and DHA can inhibit muscle mass decreases in animal [49,50,51,52]. Previous studies showed that omega-3 ingestion activated the Akt-mTOR-p70S6K pathway, which is extremely important in protein synthesis, in an animal experiment [49,52]. In addition, an investigation using dystrophin-deficit mouse found that EPA and DHA intake increased the number of activated satellite cells [53]. Similarly, DHA-enriched diet cause increases in insulin-like growth factor-1 (IGF-1) mRNA expression, Akt-mTOR-p70S6K pathway, and the fractional synthesis rate in pigs [51]. The mechanism(s) by which EPA and DHA activate IGF-1, its signals and satellite cells is (are) mostly unknown. It is speculated that unknown signal cascades that affects macrophages, the factor nuclear kappa B (NFkB) and membrane lipids composition may be involved [53]. Interestingly, the EPA increases phosphorylation of mTOR under stress conditions, while it did not affect underlying physiological conditions [50]. They also stated that confirmation in humans is required [50]. Certain evidence has been obtained regarding effects of EPA on maintaining the skeletal muscle in animals under wasting condition, but verification in humans is required.

Meanwhile, the effects of EPA and DHA supplementation in humans is limited. Smith et al. [54] reported that mTOR signals with hyperaminoacidemic-hyperinsulinemic clamp and muscle protein fractional synthesis rate increased after 1.86 g/day of EPA and 1.50 g/day of DHA for eight weeks in nine subjects (men and women aged 25–45 years). Recently, they found that supplementation of 1.86 g/day of EPA and 1.50 g/day of DHA for six months to 44 elderly subjects (omega-3 *n* = 40, control *n* = 20, men and women aged 60–85 years) caused an increase in thigh muscle mass, suggesting that this could be a new therapy for preventing sarcopenia [55]. However, these results are not consistent with the results of training experiments [56]. The 3.5 g/day of EPA and 0.9 g/day of DHA for eight weeks supplementation did not change myofibrillar muscle protein synthesis and did not decrease in protein synthesis before and after high intensity exercise in trained 20 men (21–24 years) [56]. These discrepancies were attributed to: (1) method of the protein synthesis rate; (2) method of amino acid administration; and (3) training experience of subjects. In addition, gender, age, and ingestion period may have influenced the results. Da Boit [57] investigated 2.1 g/day of EPA and 0.6 g/day of DHA for 12 weeks intake in elderly individuals (men: 70.6 ± 4.5 years, women: 70.7 ± 3.3 years). They found no effects on results such as muscle CSA, myofibrillar muscle protein synthesis rate, or p70s6k after resistance training (twice per week). In summary, the role of EPA and DHA in muscle mass under training is unclear based on the previous results, but the evidence demonstrates positive effects under wasting condition.

### 3.2. Muscle Function

Previously, muscle function is evaluated by static and dynamic torque, 1RM, rate of torque development (RTD), electromyography (EMG), electrical mechanical delay (EMD), and short physical performance tests such as balance and walking. As mentioned above, not only increased thigh muscle mass, but also increased handgrip strength and 1RM squats with 1.86 g/day of EPA and 1.50 g/day of DHA for six months in 44 elderly individuals (60–85 years) [55]. In addition, Lewis et al. [58] investigated acute training response in young trained men who ingested 0.375 g/day of EPA and 0.51 g/day of DHA for 21 days. They reported that the ingestion of EPA and DHA increased vastus lateralis EMG compared with placebo. Rodacki et al. [59] divided elderly women (45 women, age: 64 ± 1.4 years) into three groups and investigated the relationship between ~0.4 g/day of EPA and 0.3 g/day of DHA intake and training effects over 12 weeks. One group performed resistance training only for 90 days, but the other groups performed the same training with EPA and DHA supplementation for 90 or 150 days (supplemented 60 days before training). They found peak torque, RTD, EMG and EMD improved significantly in supplemental groups. The 2.1 g/day of EPA and 0.6 g/day of DHA supplementation for 18 weeks with muscle resistance training caused increases in maximal isometric torque of knee extensors, maximal isometric torque of knee extensor per CSA in elderly women, but not in elderly men [57]. Thus, in terms of muscle function, it can be possible to conclude that EPA and DHA are effective for neuromuscular adaptation after training. One possible mechanism for this effect is an increase in the incorporation of omega-3 fatty acids in the cells, particularly in the nerve and muscle [60], which results in improvement in the fluidity of the membrane and acetylcholine sensitivity [21,59,61,62]. The exact biological mechanisms underlying the beneficial effect of EPA and DHA on muscle and neuron are unknown, but EPA and DHA appear to play an important role in the adaptation. Further research is required to elucidate the effect of EPA and DHA for neuromuscular adaptation with training.

## 4. Summary and Future Directions

The main points of this review can be summarized as follows.

1. Some positive effects of EPA and DHA have been observed on ECC-induced nerve and muscle damage (muscle strength deficit, DOMS, reduced ROM, and muscle swelling), while some results are not consistent.
2. EPA and DHA may have positive effects on muscle mass under wasting condition, but it is unclear with regard to training.
3. EPA and DHA have positive effects on muscle function, especially for neuromuscular adaptation.

As mentioned above, it can be concluded that EPA and DHA have several positive roles for exercise damage and function. Unfortunately, currently, there are no clarified optimal periods and dosages for EPA and DHA. Therefore, it is necessary to investigate appropriate conditions considering age, sex, exercise experience, diseases, etc. in the future. Ingestion for 30–60 days is needed to result in uptake into the human myocardial membrane [37], while ingestion for 3–4 months increased RBC deformability in patients with angina and claudication [63,64]. Regarding dosage, it should be noted that the amount of EPA and DHA is limited to a total of 3 g per day for safety in humans by the natural medicines comprehensive database [65]. Simopoulos mentioned that most athletes, especially at the leisure level, should include in their diet EPA and DHA of about 1–2 g/day as general guidelines. Otherwise, it has been suggested that an ingestion ratio of EPA to DHA of approximately 2:1 may be beneficial in counteracting exercise-induced inflammation and for the overall health of an athlete [19,66]. In particular, the ingestion of single EPA or DHA did not elicit attenuation in several muscle damage markers. Hence, we speculate that EPA and DHA might have different roles, and thereby simultaneous ingestion of EPA and DHA has a possible synergistic effect. In the future, varied ingestion periods, doses, of either EPA or DHA, or the synergistic effects of the simultaneous ingestion of both, need to be investigated in other interventions including muscle and nerve damage, and training.

## Figures and Tables

**Table 1:** Summary of the effects of EPA/DHA supplementation on muscle strength deficit.

Reference (year)	Population (Age)	Dose (Per Day)	Duration	Exercise	Outcome
Houghton and Onambele (2012) [34]	17 healthy females (20.4 ± 2.3 years)	0.36 g EPA	3 weeks	Resistance exercise (leg flexions, leg extensions, straight leg dead lifts, walking lunges; 3 set of 10 repetitions at 70%1RM)	Ineffective
DiLorenzo et al. (2014) [35]	41 healthy, untrained males (21.8 ± 2.7 years)	2.0 g DHA	4 weeks	Elbow flexor eccentric contractions (6 sets of 10 repetitions at 140%1RM using dumbbell)	Ineffective
Lenn et al. (2002) [36]	13 males (22.7 ± 3.9 years) and 9 females (24.5 ± 5.5 years)	0.287 g EPA and 0.194 g DHA	30 days	Elbow flexor eccentric contractions (50 maximal effort at a 90 °/s using the Kin-Com dynamometer)	Ineffective
Gray et al. (2014) [38]	20 healthy, untrained males (23.0 ± 2.3 years)	1.30 g EPA and 0.30 g DHA	6 weeks	Knee extensor eccentric contractions (20 sets of 10 repetitions at a 0.52 rads/s using the Biodex isokinetic dynamometer)	Ineffective
Tsuchiya et al. (2016) [20]	24 healthy, untrained males (19.5 ± 0.8 years)	0.60 g EPA and 0.26 g DHA	8 weeks	Elbow flexor eccentric contractions (6 sets of maximal 5 repetitions at a 30 °/s using the Biodex isokinetic dynamometer)	Effective
Ochi et al. (2017) [29]	21 healthy, untrained males (21.0 ± 0.8 years)	0.60 g EPA and 0.26 g DHA	8 weeks	Elbow flexor eccentric contractions (6 sets of 10 repetitions at 40%1RM, 30 °/s using dumbbell)	Effective

EPA, eicosapentaenoic acid; DHA, docosahexaenoic acid.

**Table 2:** Summary of the effects of EPA/DHA supplementation on delayed onset muscle soreness.

Reference (Year)	Population (Age)	Dose (Per Day)	Duration	Exercise	Outcome
Houghton and Onambele (2012) [34]	17 healthy females (20.4 ± 2.3 years)	0.36 g EPA	3 weeks	Resistance exercise (leg flexions, leg extensions, straight leg dead lifts, walking lunges; 3 set of 10 repetitions at 70%1RM)	Ineffective
Lembke et al. (2014) [28]	64 healthy, untrained males and females (over the age of 18 years)	2.70 g EPA and DHA	30 days	Elbow flexor eccentric contractions (2 sets of 30 maximal efforts using the Cybex isokinetic dynamometer)	Effective
DiLorenzo et al. (2014) [35]	41 healthy, untrained males (21.8 ± 2.7 years)	2.0 g DHA	4 weeks	Elbow flexor eccentric contractions (6 sets of 10 repetitions at 140%1RM using dumbbell)	Ineffective
Lenn et al. (2002) [36]	13 males (22.7 ± 3.9 years) and 9 females (24.5 ± 5.5 years)	0.287 g EPA and 0.194 g DHA	30 days	Elbow flexor eccentric contractions (50 maximal efforts at a 90 °/s using the Kin-Com dynamometer)	Ineffective
Gray et al. (2014) [38]	20 healthy, untrained males (23.0 ± 2.3 years)	1.30 g EPA and 0.30 g DHA	6 weeks	Knee extensor eccentric contractions (20 sets of 10 repetitions at a 0.52 rads/s using the Biodex isokinetic dynamometer)	Ineffective
Tartibian et al. (2009) [31]	27 healthy males (33.4 ± 4.2 years)	0.324 g EPA and 0.216 g DHA	30 days	40-minite bench stepping (knee height step-50 cm on average-at a rate of 15 steps per minute)	Effective
Jouris et al. (2011) [27]	3 males and 8 females (18 to 60 years)	2.0 g EPA and 1.0 g DHA	2 weeks	Elbow flexor eccentric contractions (2 sets to failure at 120%1RM using dumbbell)	Effective
Tinsley et al. (2016) [33]	19 healthy, untrained females (22.5 ± 1.8 years)	3.60 g EPA and DHA	2 weeks	Elbow flexor and leg extensor eccentric contractions (10 sets to failure at 50%1RM using the elbow flexions and leg extensions machines)	Effective
Tsuchiya et al. (2016) [20]	24 healthy, untrained males (19.5 ± 0.8 years)	0.60 g EPA and 0.26 g DHA	8 weeks	Elbow flexor eccentric contractions (6 sets of maximal 5 repetitions at a 30 °/s using the Biodex isokinetic dynamometer)	Effective
Ochi et al. (2017) [29]	21 healthy, untrained males (21.0 ± 0.8 years)	0.60 g EPA and 0.26 g DHA	8 weeks	Elbow flexor eccentric contractions (6 sets of 10 repetitions at 40%1RM, 30 °/s using dumbbell)	Effective
Phillips et al. (2003) [30]	40 healthy, untrained males (18–35 years)	0.80 g DHA	2 weeks	Elbow flexor eccentric contractions (3 sets of 10 repetitions using 80% using the arm curl machine)	Ineffectiv
Bloomer et al. (2009) [26]	14 recreational males (25.5 ± 4.8 years)	2.224 g EPA and 2.208 g DHA	6 weeks	60-min treadmill climb using a weighted pack (weight equal to 25% of body mass)	Ineffective

EPA, eicosapentaenoic acid; DHA, docosahexaenoic acid.

**Table 3:** Summary of the effects of EPA/DHA supplementation on range of motion.

Reference (Year)	Population (Age)	Dose (Per Day)	Duration	Exercise	Outcome
Lembke et al. (2014) [28]	64 healthy, untrained males and females (over the age of 18 years)	2.70 g EPA and DHA	30 days	Elbow flexor eccentric contractions (2 sets of 30 maximal efforts using the Cybex isokinetic dynamometer)	Ineffective
Lenn et al. (2002) [36]	13 males (22.7 ± 3.9 years) and 9 females (24.5 ± 5.5 years)	0.287 g EPA and 0.194 g DHA	30 days	Elbow flexor eccentric contractions (50 maximal efforts at a 90 °/s using the Kin-Com dynamometer)	Ineffective
Tartibian et al. (2009) [31]	27 healthy males (33.4 ± 4.2 years)	0.324 g EPA and 0.216 g DHA	30 days	40-min bench stepping (knee height step-50 cm on average-at a rate of 15 steps per minute)	Effective
Tsuchiya et al. (2016) [20]	24 healthy, untrained males (19.5 ± 0.8 years)	0.60 g EPA and 0.26 g DHA	8 weeks	Elbow flexor eccentric contractions (6 sets of maximal 5 repetitions at a 30 °/s using the Biodex isokinetic dynamometer)	Effective
Ochi et al. (2017) [29]	21 healthy, untrained males (21.0 ± 0.8 years)	0.60 g EPA and 0.26 g DHA	8 weeks	Elbow flexor eccentric contractions (6 sets of 10 repetitions at 40%1RM, 30 °/s using dumbbell)	Effective

EPA, eicosapentaenoic acid; DHA, docosahexaenoic acid.

**Table 4:** Summary of effects of EPA/DHA supplementation on muscle swelling.

Reference (Year)	Population (Age)	Dose (Per Day)	Duration	Exercise	Outcome
Tartibian et al. (2009) [31]	27 healthy males (33.4 ± 4.2 years)	0.324 g EPA and 0.216 g DHA	30 days	40-minute bench stepping (knee height step-50 cm on average-at a rate of 15 steps per minute)	Circumference; Effective
Jouris et al. (2011) [27]	3 males and 8 females (18 to 60 years)	0.20 g EPA and 0.10 g DHA	2 weeks	Elbow flexor eccentric contractions (2 sets to failure at 120%1RM using dumbbell)	Circumference; Ineffective
Tinsley et al. (2016) [33]	19 healthy, untrained females (22.5 ± 1.8 years)	3.60 g EPA and DHA	2 weeks	Elbow flexor and leg extensor eccentric contractions (10 sets to failure at 50%1RM using the elbow flexion and leg extension machines)	Circumference; Ineffective
Tsuchiya et al. (2016) [20]	24 healthy, untrained males (19.5 ± 0.8 years)	0.60 g EPA and 0.26 g DHA	8 weeks	Elbow flexor eccentric contractions (6 sets of maximal 5 repetitions at a 30 °/s using the Biodex isokinetic dynamometer)	Circumference; Ineffective
Ochi et al. (2017) [29]	21 healthy, untrained males (21.0 ± 0.8 years)	0.60 g EPA and 0.26 g DHA	8 weeks	Elbow flexor eccentric contractions (6 sets of 10 repetitions at 40%1RM, 30 °/s using dumbbell)	Circumference; Ineffective Cross-sectional area; ineffective

EPA, eicosapentaenoic acid; DHA, docosahexaenoic acid.

**Table 5:** Summary of Effects of EPA/DHA supplementation on serum cytokines and muscle damage markers.

Reference (Year)	Population (Age)	Dose (Per Day)	Duration	Exercise	Outcome
Houghton and Onambele (2012) [34]	17 healthy females (20.4 ± 2.3 years)	0.36 g EPA	3 weeks	Resistance exercise (leg flexions, leg extensions, straight leg dead lifts, walking lunges; 3 set of 10 repetitions at 70%1RM)	CK; Ineffective IL-6; Effective
DiLorenzo et al. (2014) [35]	41 healthy, untrained males (21.8 ± 2.7 years)	2.0 g DHA	4 weeks	Elbow flexor eccentric contractions (6 sets of 10 repetitions at 140%1RM using dumbbell)	CK; Effective IL-6; Effective
Gray et al. (2014) [38]	20 healthy, untrained males (23.0 ± 2.3 years)	1.30 g EPA and 0.30 g DHA	6 weeks	Knee extensor eccentric contractions (20 sets of 10 repetitions at a 0.52 rads/s using the Biodex isokinetic dynamometer)	CK; Ineffective
Tartibian et al. (2011) [32]	45 healthy, untrained males (29.7 ± 6.6 years)	0.324 g EPA and 0.216 g DHA	30 days	40-min bench stepping (knee height step-50 cm on average-at a rate of 15 steps per minute)	CK; Effective Mb; Effective IL-6; Effective TNF-α; Effective
Jakeman et al. (2017) [39]	27 physically active males (26 ± 4 years)	High EPA group; EPA 0.75 g, DHA 0.05 g Low EPA group; EPA 0.15 g, DHA 0.10 g	One dose upon completion of the plyometric protocol	10 sets of 10 repetitions of squat jump performance and countermovement jump performance	CK; Ineffective IL-6; Ineffective
Tsuchiya et al. (2016) [20]	24 healthy, untrained males (19.5 ± 0.8 years)	0.60 g EPA and 0.26 g DHA	8 weeks	Elbow flexor eccentric contractions (6 sets of maximal 5 repetitions at a 30 °/s using the Biodex isokinetic dynamometer)	CK; Ineffective Mb; Ineffective IL-6; Effective TNF-α; Ineffective
Phillips et al. (2003) [30]	40 healthy, untrained males (18–35 years)	0.80 g DHA	2 weeks	Elbow flexor eccentric contractions (3 sets of 10 repetitions using 80% using the arm curl machine)	CK; Ineffective IL-6; Effective
Bloomer et al. (2009) [26]	14 recreational males (25.5 ± 4.8 years)	2.224 g EPA and 2.208 g DHA	6 weeks	60-min treadmill climb using a weighted pack (weight equal to 25% of body mass)	CK; Ineffective TNF-α; Effective

EPA, eicosapentaenoic acid; DHA, docosahexaenoic acid.
